# Introducing the open biomedical map of science

**DOI:** 10.3389/frma.2023.1274793

**Published:** 2023-10-04

**Authors:** Michael Ginda, Bruce W. Herr, Katy Börner

**Affiliations:** Department of Intelligent Systems Engineering, Luddy School of Informatics, Computing, and Engineering, Indiana University, Bloomington, IN, United States

**Keywords:** science mapping, PubMed (MEDLINE), biomedical science, network visualization, scientometrics

## Abstract

This article introduces work in progress to develop a new, open biomedical map of science (OBMS) using the PubMed citation database. The new science map represents bimodal network relationships between journals and medical subject heading (MeSH) descriptors, based on a journal's articles indexed in the MEDLINE component of PubMed. We review the current efforts to use PubMed data in science of science studies and science mapping. As part of the development process, we compare the journals indexed in PubMed with journals included in the 2011 UCSD map of science to establish a baseline of disciplinary coverage of PubMed for the period 2009–2019. Journal article frequency is analyzed to establish the minimum number of citations required by a journal for inclusion in a map of science. A prototype OBMS is presented, and we discuss the strengths and weaknesses of the OBMS, as well as the next steps for using and productizing this new open map for general and free usage.

## Introduction

Many maps of science have been proposed (Leydesdorff and Rafols, 2009; Börner et al., 2012; Chen, 2017; Van Eck and Waltman, 2017; Boyack et al., 2020) that use data from commercial providers which cannot be reproduced by other teams due to data and code access restrictions. These maps are not open or freely available to use or modify, though in some cases, derivative, aggregated data is publicly available that allows others to generate science map overlays.

Currently, there is no *fair* map of science, i.e., that meets principles of findability, accessibility, interoperability, and reusability (Wilkinson et al., 2016). Developing a *fair* science map requires leveraging open datasets of scientific literature that categorizes articles using open controlled vocabularies or subject classification systems, such as the PubMed article library and the medical subject heading (MeSH term) controlled vocabulary used in the biomedical sciences. The GridaMap tool creates network visualizations of MeSH terms that allows users to explore research trends in the biomedical sciences based on user generated queries (Yang et al., 2018). The PubMed database has been used to create an open model of the biomedical sciences; visualizations of the underlying document similarity network using Leiden algorithm identified over 28,000 document clusters (Boyack et al., 2020).

This paper presents ongoing research efforts to develop a *fair* map of the biomedical sciences data collected from PubMed. It details the data uses, analyses, and visualizations run, and compares the resulting map with the 2011 UCSD map of science. We conclude with a discussion of planned map usage and next steps.

## Data

To construct a *fair* map of biomedical sciences, we used data collected from an instance of the PubMed database that is continuously updated. PubMed is a citation database maintained by the U.S. National Institutes of Health's National Library of Medicine (NIH/NLM). The database is composed of journals indexed using MeSH descriptors in MEDLINE, an open, free, full-text archive of biomedical and life sciences journals in the PubMed Central (PMC), as well as books. The database currently indexes over 21 million articles across 22,880 journals published from between 1996 and 2022. We limit our analysis and science mapping efforts to journal citation components of PubMed. The PubMed database was queried on August 16, 2022, to create three data sets: (1) a list of 18,234 journals associated with 11,990,394 articles indexed by PubMed between 2009 and 2019; (2) a list of relevant MeSH descriptors; and (3) an edge list for a bimodal network that links journals and MeSH descriptors, where the weight equals the number of articles indexed with a given descriptor (target) in a journal (source).

MeSH descriptors are a controlled vocabulary that uses a hierarchical tree structure organization that is divided into 16 branches (National Library of Medicine, 2019). The vocabulary uses an *ad-hoc* organization structure within main branches of the tree to define major conceptual areas of the biomedical sciences. Other branches are used to define concepts associated with scientific disciplinary families, social groups, geography, and publication characteristics. At present, MeSH descriptors are assigned to articles from 5,200 journals that meet MEDLINE standards of scientific quality for inclusion (National Library of Medicine, 2022).

## Methods

Journal classification data from the UCSD Map of Science (Börner et al., 2012) was used to characterize the scientific disciplines of journals indexed in PubMed and then compare the source coverage to those two datasets used to build the OBMS. To complete this analysis, a list of 18,234 unique journal titles indexed by PubMed between 2009 and 2019 was collected from the database and mapped to the UCSD Map of Science using Sci2 Tool (Börner, 2011). As part of the comparison, journals are grouped by whether a title was indexed by PubMed between 2001 and 2011, which is the period covered in UCSD Map of Science. Results of the UCSD journal classifications were validated by comparing unprocessed full journal titles to potential misidentifications across 577 journal titles. Validated results compared the overlap in journal titles used to create the UCSD map with journals indexed by PubMed to describe the disciplinary coverage of PubMed indexed journals.

**Figure 1:**
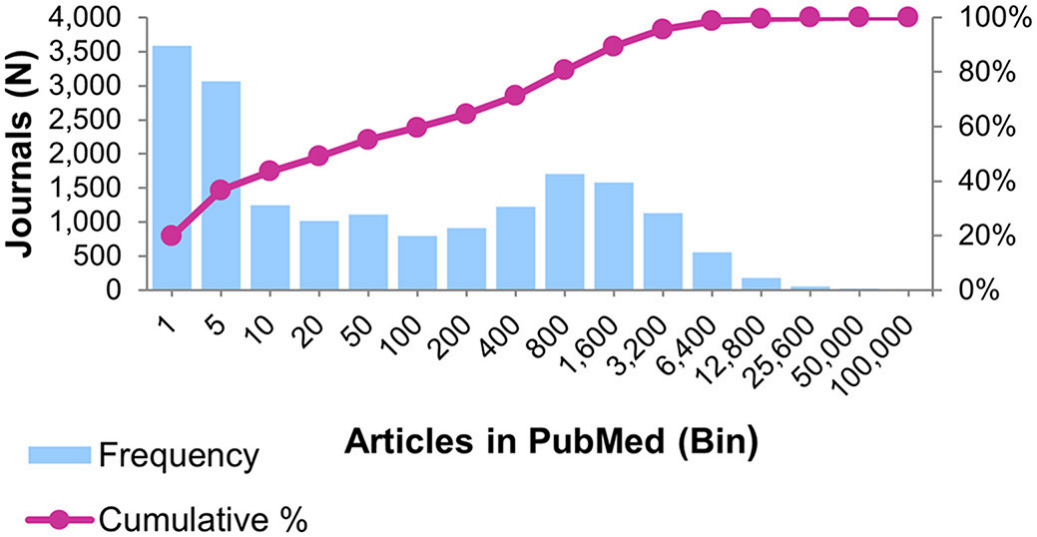
Histogram shows the distribution journals by the number of articles indexed by PubMed between 2009 and 2019.

Next, we determined the number of articles indexed in PubMed by each journal between January 2009 and December 2019 to calculate the median number of articles published per journal during this period. We then visualized the journal publication frequency distribution (see Figure 1). The result of this analysis indicates that a journal must have at least 20 articles indexed by MEDLINE for inclusion in the OBMS; 8,209 journals have the minimum article threshold.

The final version of biomedical map of science will cover the period from January 1, 2013, through December 31, 2022, and uses an undirected, weighted bimodal network to represent the relationship between journals and the MeSH descriptors associated with indexed articles. Edge weight represents the count of articles indexed under a MeSH term for a given journal. The edge list is processed to create a node list of unique journal titles and MeSH descriptors found in this network. For the initial prototyping of the science map, the edge list is filtered to keep only the top 50 MeSH descriptors linked to each journal. The filtered network is processed to identify the most important edges in the network and then identify a network layout using the Zoomable Multi-Level Tree (ZMLT) algorithm that creates map-like visualization of large network graphs (De Luca et al., 2019). The ZMLT layout has been productized as an interactive, searchable map of science using the map4sci software (https://github.com/cns-iu/map4sci).

## Results

### Comparing PubMed journals to the UCSD map of science

The PubMed citation database primarily indexes journals from the biomedical domain. However, a cursory overview of the MeSH term hierarchy tree shows that topical coverage includes many external disciplines (e.g., mathematics, physics, social, computer, and information science). The UCSD map of science incorporated data from 25,453 journals indexed by Web of Science and Scopus citation databases to provide a holistic representation of science. A comparison of the journals indexed in PubMed to those used in the UCSD map reveal an overlap of 6,307 journals, or 34.6% of the titles identified in PubMed between 2009 and 2019; results of the comparative analysis are presented in Table 1. Analysis results of the disciplinary classifications for the overlapping journal titles of UCSD map of science and PubMed show that PubMed primarily indexes journals from medical and health sciences professions as well as social sciences (see Table 2). PubMed's disciplinary coverage is weakest in the humanities, biotechnology, and earth sciences.

**Table 1:** Overlap of journal titles indexed in PubMed between 2009 and 2019 and covered in the 2011 UCSD map of science.

**UCSD 2011 mapping**	**Journals in PubMed 2001–2011**	**Journals not in PubMed 2001–2011**	**Journals (*N*)**	**Journals (%)**
Found	4,894	1,413	6,307	34.6
Not found	4,423	7,504	11,927	65.4
Journals (*N*)	9,317	8,917	18,234	
Journals (%)	51.1	48.9		100.0

**Table 2:** UCSD scientific disciplines classifications for journals indexed in PubMed, between 2009 and 2019.

**UCSD scientific disciplines**	**Journals (*N*)**	**Journals (%)**
Medical specialties	1,126	17.9
Health professionals	1,093	17.3
Social sciences	1,018	16.1
Brain research	493	7.8
Biology	457	7.2
Infectious diseases	427	6.8
Chemical, mechanical, and civil engineering	343	5.4
Chemistry	337	5.3
Electrical engineering and computer science	293	4.6
Math and physics	259	4.1
Earth sciences	170	2.7
Biotechnology	159	2.5
Humanities	132	2.1
Journals mapped to UCSD map of science	6,307	100

### Analysis of journal articles indexing in PubMed between 2009 and 2019

An analysis of articles indexed in the PubMed database between 2009 and 2019 shows that journals have a median of 23 articles published during this period. There were 77 journals that published over 25,000 articles in this 10-year time frame, with PLoS One indexing over 231,000 articles in PubMed. The distribution of journals by the frequency of indexed articles is visualized in a binned bar graph in Figure 1, with the cumulative percentage of journals plotted as a line over the bar graph. The graph shows that a little over 7,900 journals indexed 10 or fewer articles in PubMed between 2009 and 2019, or 43.45% of journals identified during the period.

### Visualizing a prototype biomedical map of science

An initial bimodal network that represents the relationships between PubMed journals and MeSH descriptors was extracted. The network includes 15,033 nodes that represent 8,209 journal nodes and 6,824 MeSH descriptors; the network includes 98,439 weighted edges. The MST Pathfinder Network Scaling algorithm was applied to this network to identify the network backbone that contains the most important edges (Quirin et al., 2008) using the Sci2 Tool implementation. The initial prototype network maintains 14,982 nodes (8,178 journal and 6,824 MeSH nodes) and 14,981 edges; 51 isolate journal nodes were created in this process and removed from the prototype map of science.

**Figure 2:**
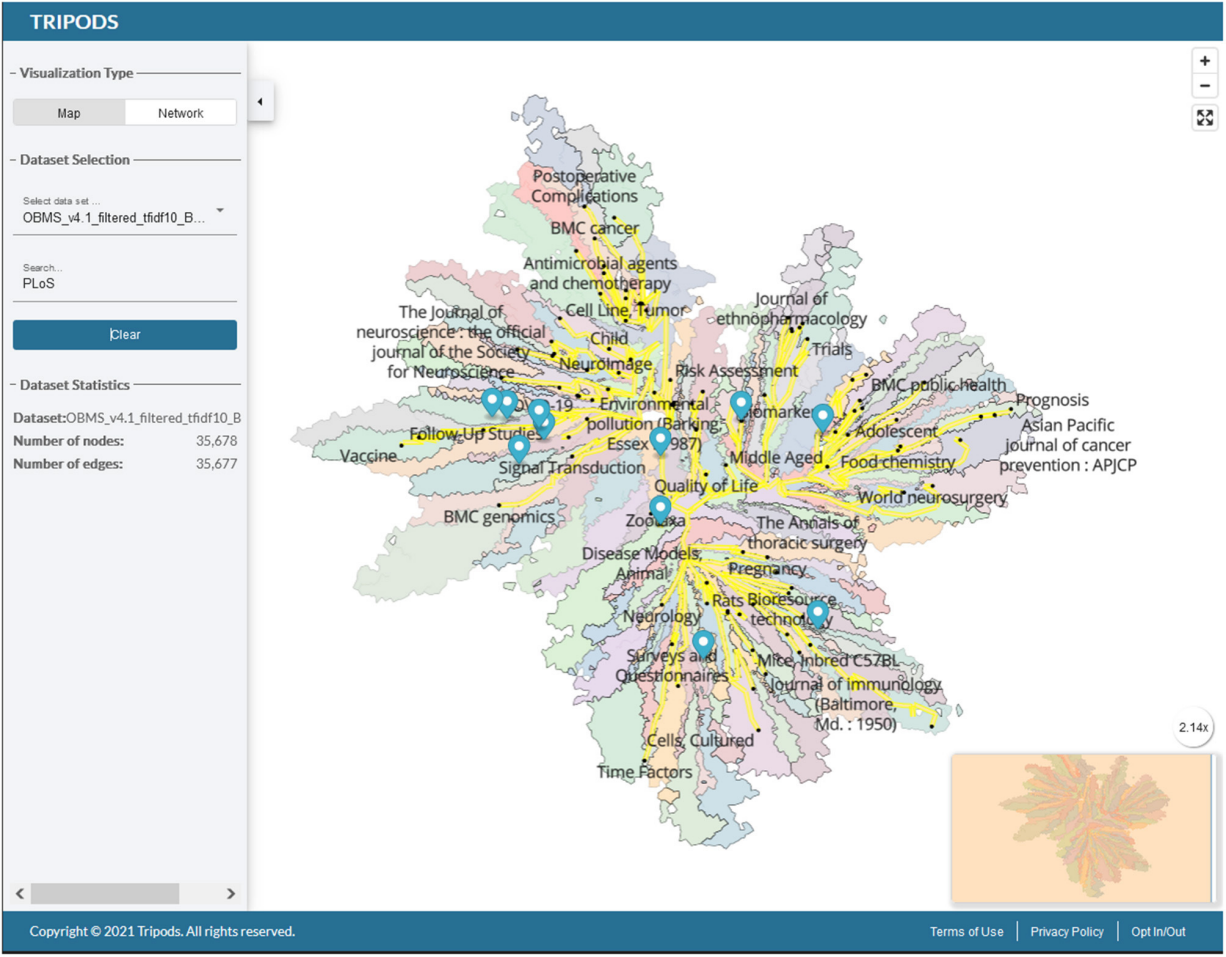
Network visualization for the open biomedical map of science (https://cns-iu.github.io/obms/visualizer).

After initial prototyping work, our team refined the underlying data sets to include 10,647,711 articles indexed in MEDLINE during the period 2013–2022. The change in temporal period allows the map to better represent the current scientific literature. The resulting networks includes 35,678 nodes (5,587 journals, and 30,091 MeSH nodes) and 35,677 edges. All resulting OBMS prototype networks are visualized as interactive networks using map4sci infrastructure (see Figure 2 and https://cns-iu.github.io/obms/).

## Discussion

Regions of the map represent node-link-groups of journals and MeSH descriptors that are closely related to each other based on the network's local neighborhoods (Saket et al., 2015; De Luca et al., 2019). The MeSH descriptors and network backbone (yellow highlights) are overlaid on the map overview to help orient viewers who are exploring the map. The prototype biomedical map of science shows a significant split between medical and health sciences with psychology and brain sciences, which branches off in a peninsula in the lower left quadrant of the map. Users can also explore the map by searching for specific journal families and MeSH descriptors, such as “PLoS,” which are marked by blue pins in Figure 2. Given that the map shows MeSH descriptor labels in addition to journals, descriptive descriptors exist for each major area of the map and labels increase in numbers and specificity when users zoom into the map. The regions were computed using k-means clustering on the full network layout. Unlike the 2011 UCSD map of science, where nodes represent 554 subdisciplines of science, the shaded regions on the prototype map are not classified to specific disciplines and subdisciplines of science.

An initial prototype visualization revealed challenges posed by MeSH geographic descriptors and named groups. For instance, country names are distributed throughout network (e.g., “Spain” and “India”), and located near the research journals and subjects' areas pursued in each geographic location or community. Similarly, MeSH descriptors for named groups (e.g., humans, male, female, etc.) and publication types add descriptors that collect many links while adding little value to a map intended to represent areas and disciplines of scientific research. In the latest iterations of the OBMS, geographic descriptors have been filtered out of the networks, and term frequency-inverse document frequency (TF-IDF) analysis is applied to the network edge list to evaluate and rank relationships that are most representative of the articles published in a journal during the period 2013–2022. Results of the TF-IDF analysis are ranked, filtered, and then processed by the MST Pathfinder Network Scaling algorithm, which determines the final set of edge included in prototype OBMS networks (see Figure 2).

All versions of prototype OBMS are limited to journals that are indexed with MeSH descriptors, which excludes over 10,000 journal titles from our initial data set. To supplement the OBMS basemap, document similarity measures generated by Boyack et al. (2020) will be used to create a weighted, undirected journal similarity network. The final version of the OBMS will support portfolio analysis of biomedical research.

Initial work on the prototype OBMS has demonstrated feasibility of using bimodal network of journal-MeSH descriptor links to create a map of the biomedical sciences. Future development will take a two-step process to (1) create a basemap using 8,209 journals and high TF-IDF MeSH descriptors, and (2) to use document clustering results generated by Boyack et al. (2020) to supplement the basemap with additional journals indexed in PubMed Commons that lack MeSH descriptor indexing. Additionally, we will present an example of portfolio analysis of biomedical research projects, such as the Human BioMolecular Atlas Program (HuBMAP).

## Data availability statement

The original contributions presented in the study are included in the article/Supplementary material, further inquiries can be directed to the corresponding author.

## Author contributions

MG: Conceptualization, Data curation, Formal analysis, Methodology, Project administration, Writing—original draft, Writing—review and editing. BH: Data curation, Methodology, Project administration, Resources, Software, Visualization, Writing—review and editing. KB: Conceptualization, Funding acquisition, Resources, Supervision, Writing—review and editing.
